# The Annual Commencement of the University of Maryland, Dental Department

**Published:** 1886-02

**Authors:** 


### Editorial, Etc.

---

THE ANNUAL COMMENCEMENT OF THE UNIVERSITY OF MARYLAND, DENTAL DEPARTMENT, will be held in the Academy of Music, Baltimore, on Wednesday, March 17th, 1886. Although the graduating class will be smaller in number than those of previous years, yet the number of matriculates during the present session of 1885-86, is larger than ever before. A strict compliance with the resolution adopted by the "Association of State Dental Examiners," and the "American Dental Association," in regard to the requirement of *two full sessions of five months each before graduation*, is the cause of a smaller graduating class this year than usual, as no less than *twenty-two* applicants for graduation after one session's attendance and five and more years of practice, were refused; otherwise the graduating class of the present session would have been larger than those of other years, instead of smaller.

In the graduating class of the year 1885, no less than 17 were one session students, who were admitted to the senior class on five and more years of dental practice, which heretofore has been regarded as equivalent to one session's attendance. The University of Maryland Dental Department is endeavoring to maintain the highest standard of dental education possible and will always be found ready to second the efforts of State Dental Examining Boards and Dental Associations in advancing the requirements for graduation to a proper grade. And although over twenty applicants have this session been refused graduation, yet in the end the Faculty are satisfied that their efforts in this direction will be appreciated by the profession. The fact that this Department has had during the present session of 1885-86 its largest class of students, is proof positive of its reputation as a dental school. Hence the sacrifice was willingly made in order to comply with the requirements for graduation as adopted by the several Associations. The question may also be asked: "How many of the Colleges belonging to the Association of Dental Faculties have followed the example of the University of Maryland Dental Department during the present session of 1885-86. in regard to the *two sessions rule?*"

The printed proceedings of the Association of Dental Faculties plainly show that this rule of two sessions was to go into effect in June, 1885, and that all members of said association had obligated themselves to conform to it. How it can be construed to apply to future sessions and not to the present ones is beyond comprehension, if the printed proceedings of the meetings are correct. However, there is no question concerning the strict compliance with said rule on the part of the University of Maryland.

